# Comparison of BinaxNOW and SARS-CoV-2 qRT-PCR Detection of the Omicron Variant from Matched Anterior Nares Swabs

**DOI:** 10.1128/spectrum.01307-22

**Published:** 2022-10-18

**Authors:** Lena Landaverde, Jacquelyn Turcinovic, Lynn Doucette-Stamm, Kevin Gonzales, Judy Platt, John H. Connor, Catherine Klapperich

**Affiliations:** a Department of Biomedical Engineering, Boston University, Boston, Massachusetts, USA; b Clinical Testing Laboratory, Boston University, Boston, Massachusetts, USA; c Precision Diagnostics Center, Boston University, Boston, Massachusetts, USA; d Department of Microbiology, Boston University School of Medicine, Boston, Massachusetts, USA; e National Emerging Infectious Diseases Laboratories, Boston University, Boston, Massachusetts, USA; f Program in Bioinformatics, Boston University, Boston, Massachusetts, USA; g Student Health Services, Healthway, Boston University, Boston, Massachusetts, USA; h Office of Research, Boston University, Boston, Massachusetts, USA; i Center for Emerging Infectious Disease Research and Policy, Boston University, Boston, Massachusetts, USA; Keck School of Medicine of the University of Southern California

**Keywords:** SARS-CoV-2, COVID-19, rapid diagnostic tests, Omicron, antigen, anterior nares, BinaxNow, RT-PCR

## Abstract

The COVID-19 pandemic has increased use of rapid diagnostic tests (RDTs). In winter 2021 to 2022, the Omicron variant surge made it apparent that although RDTs are less sensitive than quantitative reverse transcription-PCR (qRT-PCR), the accessibility, ease of use, and rapid readouts made them a sought after and often sold-out item at local suppliers. Here, we sought to qualify the Abbott BinaxNOW RDT for use in our university testing program as a method to rule in positive or rule out negative individuals quickly at our priority qRT-PCR testing site. To perform this qualification study, we collected additional swabs from individuals attending this site. All swabs were tested using BinaxNOW. Initially as part of a feasibility study, test period 1 (*n* = 110) samples were stored cold before testing. In test period 2 (*n* = 209), samples were tested immediately. Combined, 102/319 samples tested severe acute respiratory syndrome coronavirus 2 (SARS-CoV-2) positive via qRT-PCR. All sequenced samples were Omicron (*n* = 92). We calculated 53.9% sensitivity, 100% specificity, a 100% positive predictive value, and an 82.2% negative predictive value for BinaxNOW (*n* = 319). Sensitivity would be improved (75.3%) by changing the qRT-PCR positivity threshold from a threshold cycle (*C_T_*) value of 40 to a *C_T_* value of 30. The receiver operating characteristic (ROC) curve shows that for qRT-PCR-positive *C_T_* values of between 24 and 40, the BinaxNOW test is of limited value diagnostically. Results suggest BinaxNOW could be used in our setting to confirm SARS-CoV-2 infection in individuals with substantial viral load, but a significant fraction of infected individuals would be missed if we used RDTs exclusively to rule out infection.

**IMPORTANCE** Our results suggest BinaxNOW can rule in SARS-CoV-2 infection but would miss infections if RDTs were exclusively used.

## INTRODUCTION

Experience in the winter of 2020 suggested that 2021 fall/winter holiday travel would also lead to an increase in severe acute respiratory syndrome coronavirus 2 (SARS-CoV-2) positivity rates (1). This prediction was confirmed when holiday travel coupled with the emergence of the WHO-defined Omicron variant (PANGO lineage B.1.1.529) initiated unprecedented levels of infection beginning in December of 2021. Omicron accounted for most cases in the United States a few weeks after it was first detected in the United States on 1 December 2021 (2–4). The Omicron surge overwhelmed many existing quantitative reverse transcription-PCR (qRT-PCR) diagnostic sites, driving an increased use of rapid diagnostic tests (RDTs). These tests rely on viral antigen detection and were developed to recognize SARS-CoV-2 variants that existed before the appearance of the highly mutated Omicron lineage.

The Abbott BinaxNOW COVID-19 antigen self-test (BinaxNOW; Abbott, Des Plaines, IL) (nucleocapsid protein target) has been at the forefront of rapid diagnostic testing in the United States. However, the debate about effectiveness of these tests in different use cases has led to efforts to track their specificity and sensitivity as new variants emerge (5–10). At the time of this study, there are an Omicron limit of detection (LOD) dilution study (11) and a selective study focused on reported lower limits of detection (LLOD) threshold cycle (*C_T_*) value ranges for BinaxNOW (12). These studies suggested that there was analytical strength in the RDT, but also suggested a limited range of viral loads in which the assays consistently returned positive results from PCR-positive samples. Two additional studies look at a broader range of SARS-CoV-2 RDTs, including a 15-day study of individuals with no symptoms comparing WHO-defined Delta (PANGO lineages B.1.617.2/AY.X) and Omicron on 3 RDTs (13) and a study testing previously PCR-tested frozen samples on 5 different RDTs (14).

In January and February of 2022, the Boston University Clinical Testing Laboratory (BU CTL) was testing all members of the BU campus community at least once a week using qRT-PCR as reported previously (15, 16). Symptomatic individuals, previously positive individuals scheduled for follow on testing, or those deemed close contacts can elect to take a test at any time and are directed to a special priority testing site at the BU Health Services Annex. As a continuing improvement exercise aimed at reducing cost and speeding turnaround time for testing at this site, we investigated whether the BinaxNOW test could be used effectively. The investigation focused on whether RDT use could provide a rapid rule-in or rule-out of SARS-CoV-2 infection in this population.

To assess the differential sensitivity for SARS-CoV-2 detection between our qRT-PCR test and BinaxNOW RDTs, we collected an additional swab from a series of individuals attending our test site described above. These extra swabs were tested using BinaxNOW tests. Two sets of samples for RDT testing were collected during test period 1 and test period 2. The first set of additional swabs were collected and refrigerated, and all were tested at the end of the study period, while a second (and larger) set of additional swabs were tested on site with no storage within 15 min of collection. The results from these matched swabs were compared to qRT-PCR results. All positive samples were sequenced to determine the SARS-CoV-2 variant.

## RESULTS AND DISCUSSION

**FIG 1:**
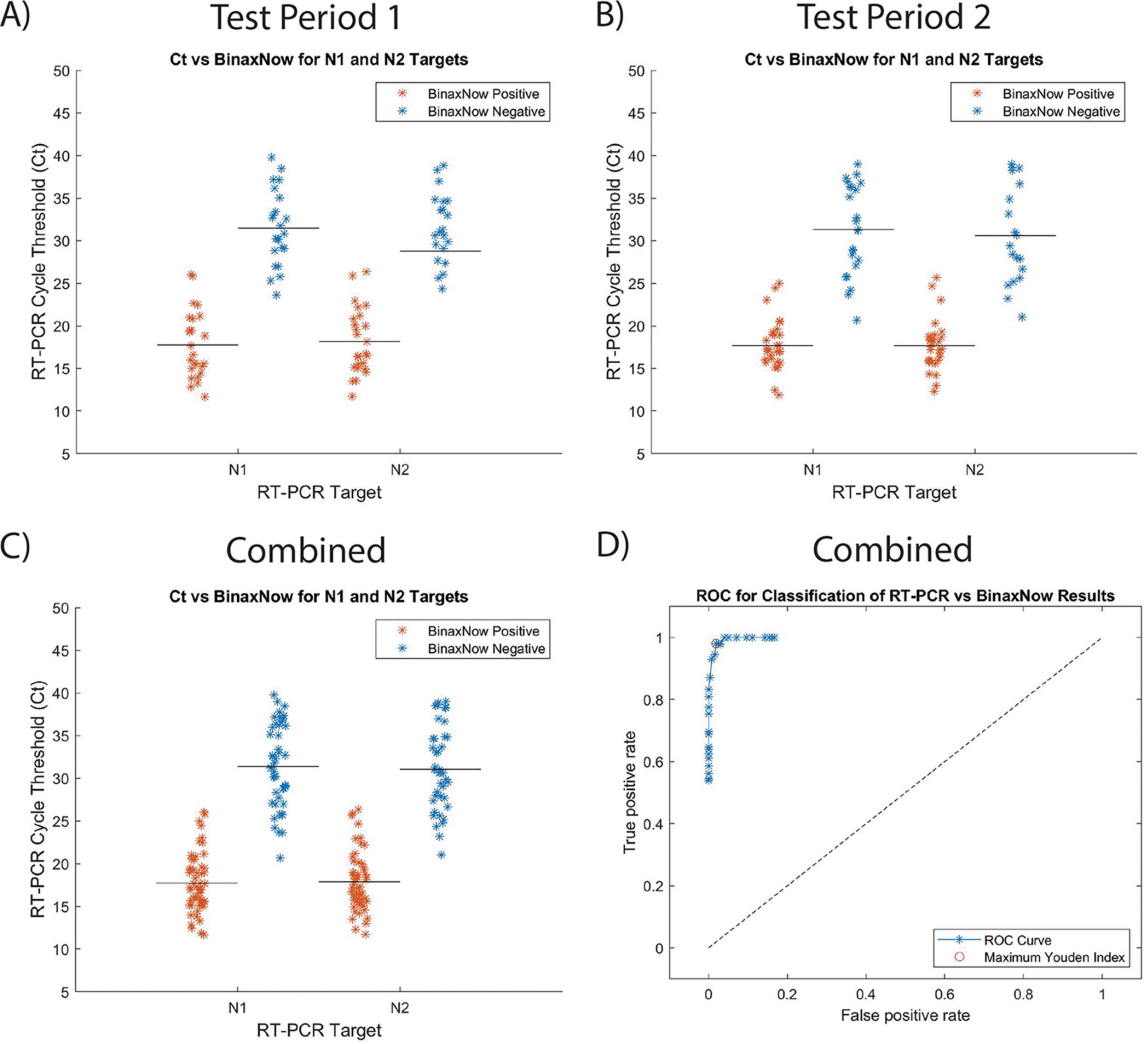
Plots showing the qRT-PCR-positive sample *C_T_* values by target. (A) In test period 1, the BinaxNOW RDTs were positive for *C_T_* values from 11.7 to 26.0 for N1 and 11.7 to 26.3 for N2. The BinaxNOW RDT-negative tests had *C_T_* values from 23.6 to 39.8 for N1 and 24.3 to 38.8 for N2. The average *C_T_* for N1 is 24.3 with a standard deviation of 8.0. The average *C_T_* for N2 is 24.2 with a standard deviation of 7.7. (B) In test period 2, the BinaxNOW RDTs were positive for *C_T_* values from 11.9 to 24.9 for N1 and 12.3 to 25.6 for N2. The BinaxNOW RDT-negative tests had *C_T_* values from 20.7 to 39.0 for N1 and 21.0 to 39.0 for N2. The average *C_T_* for N1 is 23.6 with a standard deviation of 7.9. The average *C_T_* for N2 is 22.7 with a standard deviation of 7.5. (C) The combined data set has an average *C_T_* for N1 of 23.9 with a standard deviation of 7.9. The average *C_T_* for N2 is 23.4 with a standard deviation of 7.6. (D) ROC curve for qRT-PCR versus BinaxNOW of the combined data set using qRT-PCR *C_T_* values of ≤40 as the gold standard. Within each qRT-PCR target N1 and N2, data for panels A, B, and C are summarized in Table S3.

In the first test period (10 to 12 January 2022), 48/110 matched swabs were positive for COVID-19 by the standard-of-care qRT-PCR test. The *C_T_* values for the positive samples ranged between 11.7 and 38.8 for N1 and 11.7 and 38.8 for N2 (Fig. 1A). Using the BU CTL qRT-PCR test as the “gold standard,” the BinaxNOW tests detected 25/48 positive results and 62/62 negative results (Table 1 and see Fig. S2 in the supplemental material), leading to a calculated sensitivity of 52.1% (95% confidence interval [CI], 37.4 to 66.5%), specificity of 100% (95% CI, 92.7 to 100%), a positive predictive value (PPV) of 100% (95% CI, 83.4 to 100%), and a negative predictive value (NPV) of 72.9% (95% CI, 62.0 to 81.7%).

**TABLE 1:** Two-by-two table for qRT-PCR and BinaxNOW matched samples with BU Clinical Testing Laboratory classification

Parameter^*a*^	No. (%) of samples		
	RT-PCR^+^ (*C_T_* < 40)	RT-PCR^−^ (C_T_ > 40)	Total
Test period 1			
Binax^+^	25 (22.7)	0 (0)	25 (22.7)
Binax^−^	23 (20.9)	62 (56.4)	85 (77.3)
Total	48 (43.6)	62 (56.4)	110
Test period 2			
Binax^+^	30 (14.4)	0 (0)	30 (14.4)
Binax^−^	24 (11.5)	155 (74.2)	179 (85.6)
Total	54 (25.8)	155 (74.2)	209
Combined			
Binax^+^	55	0	55
Binax^−^	47	217	264
Total	102	217	319

a In test period 1, the RDT was run after swabs were in cold storage, and in test period 2, the RDT was run in real time.

In the second test period (7 to 11 and 14 to 17 February 2022), 54/209 matched swabs were positive for COVID-19 by the qRT-PCR test. The *C_T_* values for the positive samples were between 11.8 and 39.0 for N1 and 12.2 and 39.0 for N2 (Fig. 1B). This resulted in a calculated sensitivity of 55.6% (95% CI, 41.5 to 68.9%), specificity of 100% (95% CI, 97.0 to 100%), a PPV of 100% (95% CI, 85.9 to 100%), and an NPV of 86.6% (95% CI, 80.5 to 91.1%) (Table 1 and Fig. S2).

A total of 100 positive samples had enough residual material after testing for sequencing: 92/100 were successfully sequenced and 100% (*n* = 92) were the Omicron variant (Tables S1 and S2). This result was expected, as Omicron was known to be the dominant variant in Boston during the test periods.

The mean and standard deviation were similar for both test periods (Table S3). Statistically there is no difference between the test periods for the N1 target (Kruskal-Wallis test). There was a small but significant difference between the two sample collection groups for the N2 target. The combined data set from the two study periods yielded a sensitivity of 53.9% (95% CI, 43.8 to 63.7%), specificity of 100% (95% CI, 97.8 to 100%), PPV of 100% (95% CI, 91.9 to 100%), and an NPV of 82.2% (95% CI, 76.9 to 86.5%) (Table 2). The combined *C_T_* values for positive samples range between 11.6 and 39.8 N1 and 11.7 and 39.0 N2 (Fig. 1C).

**TABLE 2:** Combined data set sensitivity with various *C_T_* cutoffs

Parameter	% Sensitivity			
	All data	BU CTL classification (*C_T_* ≤ 40)	Pilarowski et al. classification (*C_T_* ≤ 30)^*a*^	ROC curve (*C_T_* ≤ 24)
Sensitivity	53.9	53.9	75.3	94.8

a For details, see reference 5.

The sensitivity calculations depend heavily on established criteria for positivity. The cutoff for a positive result for the BU CTL qRT-PCR test is a *C_T_* value of ≤40 for one or both N1 and N2 targets. Others have advocated for using a lower *C_T_* value, reasoning individuals who have higher *C_T_* values may not transmit virus (17, 18). For example, Pilarowski, et al. use a *C_T_* cutoff of 30 for positivity (Table S4) (5). Using that cutoff for our combined data set, our revised sensitivity would be 75.3%, specificity 100%, PPV 100%, and NPV 93.2% (Table 2). In either case, the sensitivity is significantly lower than the initially published 93.3% for BinaxNOW (5).

Here, the BinaxNOW RDT returned variable test results at *C_T_* values between 20.7 and 26.0 for N1 and 21.0 to 26.3 for N2 (Fig. 1C). The mean *C_T_* value is 17.7 with a standard deviation of 3.4 for N1 and mean *C_T_* value is 17.9 with a standard deviation of 3.4 for N2 when both qRT-PCR and BinaxNOW are positive (Table S3). From the data, samples with a *C_T_* lower than 24 are highly likely to test positive on the BinaxNOW RDT. These results are consistent with a smaller study on an Omicron outbreak that paired saliva samples tested with RT-PCR and nasal rapid antigen tests (Quidel QuickVue at-home OTC COVID-19 test and Abbott BinaxNOW COVID-19 antigen self-test) (19).

Figure 1D shows a receiver operating characteristic (ROC) curve constructed for the full data set. The maximum Youden index for the ROC curve identifies where both sensitivity and specificity are at maximum (20, 21). A Youden index of 0.96 corresponds to a *C_T_* of 24 for the qRT-PCR cycle cutoff (Fig. 1D). This result provided important information regarding our ability to implement BinaxNOW antigen-based testing as a surrogate for qRT-PCR testing. A cutoff value of 24 for qRT-PCR is below the average *C_T_* value reported for both Delta and Omicron variants (5, 6, 8, 9, 12, 13), suggesting that RDT testing alone would be insufficient to maintain control of spread in our community (Table S5). Thus, the conclusion of our qualification study was that it is not possible to use the BinaxNOW test alone to replace qRT-PCR in our testing site for ruling out positive individuals. We estimate that had we only used the RDT, we would have missed 46% of the cases.

It was already known, and expected, that RDTs, like BinaxNOW, have a lower sensitivity than qRT-PCR for detecting SARS-CoV-2 (5–10). The data presented here extend this conclusion to include a real-world application where almost all the cases were confirmed to be the Omicron variant. As more data emerge linking transmission rates with viral loads, more solid conclusions will be made about the best-use cases for RDTs. Until that time, it is still prudent to be cautious about a negative BinaxNOW test, especially when symptoms are present.

Limitations of this study include the relatively small sample size compared to other studies of the BinaxNOW RDT earlier in the pandemic (6). The disease prevalence at our clinic measured by qRT-PCR on the first 2 days of testing was approaching 50% and for the second round of testing was about 25%. Recall that this site was biased toward symptomatic individuals and close contacts of known cases. The mean prevalence during the first test period in our entire population (approximately 40,000 people) was 5.0%, and in the second test period it was 0.9%.

It is also important to note that we asked individuals to swab each nostril twice, and the swab for the BinaxNOW test was always taken second. It is possible that less material was present on the swabs taken for the RDT, which could account for some of the lower sensitivity. Finally, we would like to note that 19 individuals were tested more than once, and thus, our combined data set includes 319 matched samples from 300 individuals. We do know that the 19 individuals who tested two or more times never tested positive more than once. Future research for antigen-based RDTs can provide a better understanding of what impacts the detectable range of these tests.

To be sure, tests like BinaxNOW are valuable tools, as they provide immediate results, require no instrumentation, and are highly effective at rule-in diagnosis. However, there is still an unmet need for more sensitive rapid diagnostics for SARS-CoV-2 that could augment qRT-PCR testing at times of high demand.

## MATERIALS AND METHODS

### Sample collection.

Participants in the qRT-PCR testing program were asked to give a second swab in a sequential manner as they arrived at the BU Health Services Annex for their scheduled appointment. At this site, the qRT-PCR sample is collected from the anterior nares (AN) with an ORAcollect•RNA swab (DNA Genotek, Inc., ON, Canada) (22). Our Emergency Use Authorization (EUA) application specifies use of the ORAcollect•RNA swab as a swab based on past authorization (23). Each participant provided one additional AN swab immediately after they provided their initial swab for the routine qRT-PCR test. The additional matched AN swab was taken using a Puritan sterile foam-tipped applicator (Puritan, Guilford, ME) swab that was placed into a dry, sterile 15-mL conical tube. Individuals swabbed both nostrils while observed by on-site testing personnel. Individuals testing at this location can be symptomatic, a close contact of a positive individual, or a suspected positive individual coming for a confirmatory test.

Qualification testing of the BinaxNOW RDT was first conducted on 10 and 12 January 2022. In this study, in test period 1, 110 sets of paired AN swabs were collected from 106 individuals (4 individuals tested on both dates) as observed self-collections (110 ORAcollect•RNA swabs for PCR and 110 Puritan swabs for BinaxNOW tests). Matched samples collected for the RDT were stored at the end of each collection day at 4°C, and a subset were initially tested on 13 January with the BinaxNOW RDT. Since this was a qualification study, in test period 1, we prioritized and preferentially tested the qRT-PCR SARS-CoV-2-positive samples and 5 select negative samples on 13 January. The remaining samples were all negative by qRT-PCR. The SARS-CoV-2 qRT-PCR-negative samples (Puritan swabs) were stored at −80°C until they were run on the BinaxNOW RDT on 23 January.

Later, to ensure that storing samples overnight before testing with the BinaxNOW RDT did not affect the results, we did follow-on testing on 7 to 11 February and 14 to 17 February 2022 (23). During this period, test period 2, samples were collected using the protocol described above, but the BinaxNOW RDTs were run in real time within 15 min of sample collection at the BU Health Services Annex. There was no cold storage for the swabs used for the BinaxNOW RDTs. In this test period, 209 sets of paired AN swabs were collected from 195 individuals (14 individuals tested more than once in this test period) as observed self-collections.

Altogether, 319 sets of paired swabs were collected during the two test periods from 300 individuals (19 individuals tested more than once during the combined study periods). A timeline of the two test periods details the collection, cold storage, and testing (see Fig. S1 in the supplemental material).

We received clearance from the BU Charles River Campus IRB to publish the results, as the work was ruled not human subjects research because we were evaluating the performance of the test and not accessing personal health information from the individuals (BU CRC IRB exemption no. 6402X).

### qRT-PCR testing.

ORAcollect•RNA swabs were processed, extracted, and tested by qRT-PCR at the BU CTL as detailed by Landaverde et al. (15). The BU CTL laboratory developed test detects N1 and N2 targets and RNase P as a human RNA control. All qRT-PCR tests for this work were performed individually, and none were pooled.

### Abbott BinaxNOW COVID-19 antigen self-test.

In the first test period, the additional matched AN swabs were stored as described above and subsequently tested using the manufacturer’s instructions. Briefly, 6 drops of the provided buffer were added, the swab was inserted into the BinaxNOW card and rotated 3 times clockwise, and then the card was sealed with the integrated adhesive strip. After 15 min, the test was read from the results window, and a photograph of the result was taken as a record of the test. A positive control provided in the kit was run on a separate test card to confirm the validity of the test kit. In the second test period, the additional matched AN swabs were tested at the site according to the manufacturer’s instructions within 15 min of collection.

### SARS-CoV-2 sequencing.

Whole-genome sequencing was performed on RNA extracted from all qRT-PCR-positive samples using the excess discarded ORAcollect•RNA solution. Sequencing was performed using the Illumina COVIDSeq assay (Illumina, San Diego, CA), and sequences were run on an Illumina NextSeq 500 (24). Full-length genomes for each amplified sample were then assembled through alignment to the Wuhan-Hu-1 reference sequence (NC_045512.2) (25) using Bowtie2 (26). Nucleotide substitutions, insertions, and deletions were identified with LoFreq (27) Lineage assignment for each genome was carried out using PANGO v.4.0.6 and PANGO data v.1.9 (28).

### Data analysis.

Data analysis was performed in MATLAB (MathWorks, Natick, MA). The sensitivity, specificity, positive predictive value (PPV), negative predictive value (NPV), and ROC curve were determined for the BinaxNOW RDT using the qRT-PCR test as the “gold standard.”

### Data availability.

The sequenced genomic data have been deposited in GISAID (www.gisaid.org). The data are available upon request.
