# Supplementary material for: Comparison of BinaxNOW and SARS-CoV-2 qRT-PCR Detection of the Omicron Variant from Matched Anterior Nares Swabs
Source: Microbiol Spectr. 2022 Oct 18;10(6):e01307-22. doi: 10.1128/spectrum.01307-22 (PMC9769721; doi:10.1128/spectrum.01307-22)
Supplement: Supplemental file 1 — Fig. S1 and S2 and Tables S1 to S5. Download spectrum.01307-22-s0001.pdf, PDF file, 2.7 MB [file spectrum.01307-22-s0001.pdf]

Supplemental Data:

Supplemental Figure 1. Timeline of the two test periods. The samples were collected and tested over two different test periods. All samples were collected at the BU Health Services Annex. A) All AN ORAcollect•RNA samples in Test Period 1 and 2 were stored at 4°C per the EUA approved CLIA assay requirements. B) Samples collected for the gold standard EUA approved CLIA assay were immediately processed in the BU CTL for qRT-PCR. C) As part of the initial qualification study (Test Period 1), the matched AN swabs collected for BinaxNOW™ RDTs were initially stored at 4°C until the qRT-PCR results were available. D) Remaining untested matched AN swabs negative for SARS-CoV-2 by qRT-PCR were stored in -80°C until tested on January 23. E) All of the qRT-PCR tests positive for SARS-CoV-2 and 5 select negative samples were tested in Test Period 1 on January 13. F) The samples from D were tested on January 23. G) All matched AN swabs collected during Test Period 2 were tested real-time within 15 minutes of collection on BinaxNOW™ RDTs.

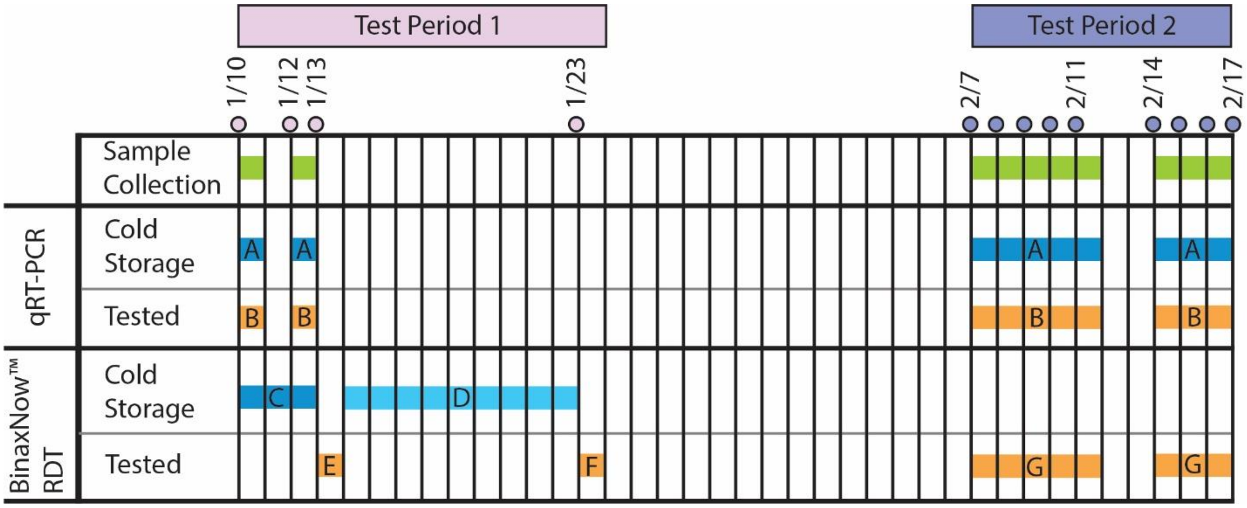

Supplemental Figure 2. Summary of data set with qRT-PCR and BinaxNOW™ RDT. Photographs of all BinaxNOW™ test cards run in this study. Tests A-1 through A-110 were performed on swabs that had been kept in cold storage for one or more days. Tests B-1 through B-209 were run immediately after swabbing at the test site. None of the images have been altered or adjusted, since these were taken only as a record of each test. They were taken with an iPhone, 15 minutes after the test was started.

Sample ID	BinaxNow™	N1 CT	N2 CT	Consensus
A-1	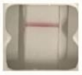	Undetermined	Undetermined	Yes
A-2	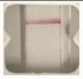	Undetermined	Undetermined	Yes
A-3	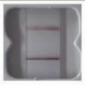	15.44	16.43	Yes
A-4	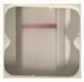	Undetermined	Undetermined	Yes
A-5	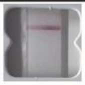	33.42	34.82	No
A-6	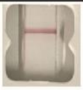	Undetermined	Undetermined	Yes
A-7	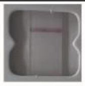	Undetermined	Undetermined	Yes
A-8	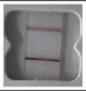	14.96	15.15	Yes
A-9	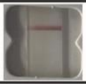	Undetermined	Undetermined	Yes
A-10	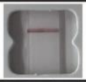	36.13	37	No
A-11	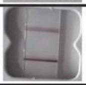	13.97	14.9	Yes
A-12	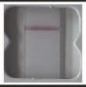	30.23	31.02	No
A-13	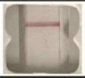	Undetermined	Undetermined	Yes
A-14	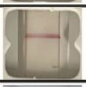	Undetermined	Undetermined	Yes
A-15	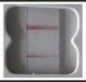	21.14	22.41	Yes
A-16	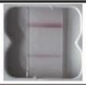	22.66	22.93	Yes
A-17	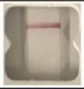	Undetermined	Undetermined	Yes
A-18	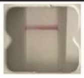	Undetermined	Undetermined	Yes
A-19	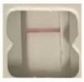	Undetermined	Undetermined	Yes
A-20	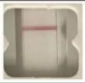	Undetermined	Undetermined	Yes
A-21	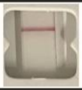	Undetermined	Undetermined	Yes
A-22	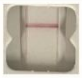	Undetermined	Undetermined	Yes
A-23	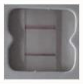	19.45	20	Yes
A-24	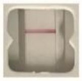	Undetermined	Undetermined	Yes
A-25	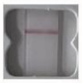	37.15	38.27	No
A-26	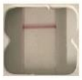	Undetermined	Undetermined	Yes
A-27	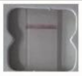	39.76	Undetermined	No
A-28	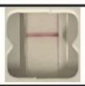	Undetermined	Undetermined	Yes
A-29	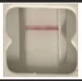	Undetermined	Undetermined	Yes
A-30	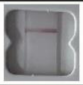	23.63	24.32	No
A-31	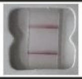	15.97	16.49	Yes
A-32	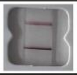	14.43	15.03	Yes
A-33	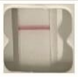	Undetermined	Undetermined	Yes
A-34	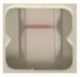	Undetermined	Undetermined	Yes
A-35	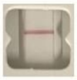	Undetermined	Undetermined	Yes
A-36	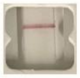	Undetermined	Undetermined	Yes

Sample ID	BinaxNow™	N1 Cт	N2 Cт	Consensus
A-37	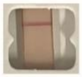	Undetermined	Undetermined	Yes
A-38	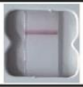	30.21	30.62	No
A-39	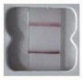	19.45	20.11	Yes
A-40	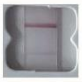	25.82	25.89	Yes
A-41	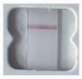	35.01	34.68	No
A-42	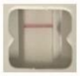	Undetermined	Undetermined	Yes
A-43	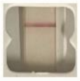	Undetermined	Undetermined	Yes
A-44	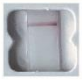	25.76	26.01	No
A-45	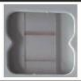	20.95	21.21	Yes
A-46	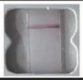	38.47	Undetermined	No
A-47	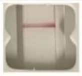	Undetermined	Undetermined	Yes
A-48	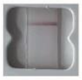	29.07	29.57	No
A-49	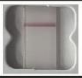	32.98	33.68	No
A-50	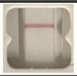	Undetermined	Undetermined	Yes
A-51	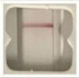	Undetermined	Undetermined	Yes
A-52	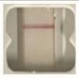	Undetermined	Undetermined	Yes
A-53	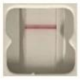	Undetermined	Undetermined	Yes
A-54	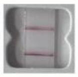	17.65	18.11	Yes
A-55	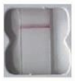	32.66	34.59	No
A-56	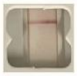	Undetermined	Undetermined	Yes
A-57	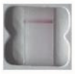	32.53	32.96	No
A-58	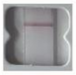	37.17	38.8	No
A-59	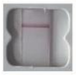	28.8	29.87	No
A-60	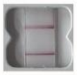	15.59	16.32	Yes
A-61	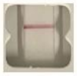	Undetermined	Undetermined	Yes
A-62	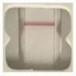	Undetermined	Undetermined	Yes
A-63	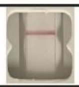	Undetermined	Undetermined	Yes
A-64	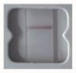	31.74	33.53	No
A-65	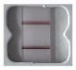	13.83	14.54	Yes
A-66	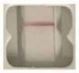	Undetermined	Undetermined	Yes
A-67	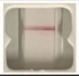	Undetermined	Undetermined	Yes
A-68	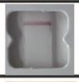	26.96	27.32	No
A-69	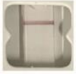	Undetermined	Undetermined	Yes
A-70	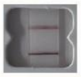	15.24	15.52	Yes
A-71	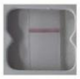	Undetermined	Undetermined	Yes
A-72	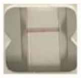	30.81	31.31	No

Sample ID	BinaxNow™	N1 CT	N2 CT	Consensus
A-73	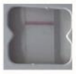	29.18	29.1	No
A-74	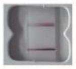	12.84	13.55	Yes
A-75	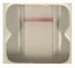	Undetermined	Undetermined	Yes
A-76	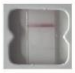	20.8	20.8	Yes
A-77	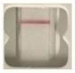	Undetermined	Undetermined	Yes
A-78	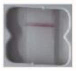	26.96	27.68	No
A-79	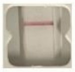	Undetermined	Undetermined	Yes
A-80	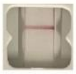	Undetermined	Undetermined	Yes
A-81	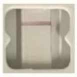	Undetermined	Undetermined	Yes
A-82	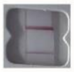	26.02	26.34	Yes
A-83	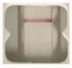	Undetermined	Undetermined	Yes
A-84	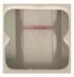	Undetermined	Undetermined	Yes
A-85	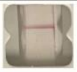	Undetermined	Undetermined	Yes
A-86	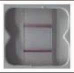	18.83	18.97	Yes
A-87	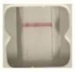	Undetermined	Undetermined	Yes
A-88	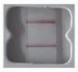	15.56	15.6	Yes
A-89	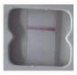	30.13	30.58	No
A-90	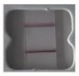	16.61	16.71	Yes

Sample ID	BinaxNow™	N1 CT	N2 CT	Consensus
A-91	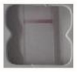	25.29	25.58	No
A-92	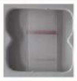	19.31	19.54	Yes
A-93	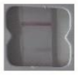	22.43	22.2	Yes
A-94	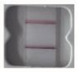	11.67	11.73	Yes
A-95	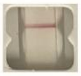	Undetermined	Undetermined	Yes
A-96	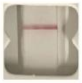	Undetermined	Undetermined	Yes
A-97	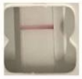	Undetermined	Undetermined	Yes
A-98	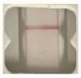	Undetermined	Undetermined	Yes
A-99	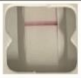	Undetermined	Undetermined	Yes
A-100	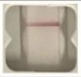	Undetermined	Undetermined	Yes
A-101	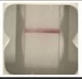	Undetermined	Undetermined	Yes
A-102	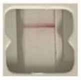	Undetermined	Undetermined	Yes
A-103	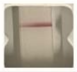	Undetermined	Undetermined	Yes
A-104	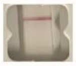	Undetermined	Undetermined	Yes
A-105	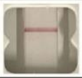	Undetermined	Undetermined	Yes
A-106	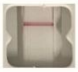	Undetermined	Undetermined	Yes
A-107	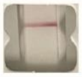	Undetermined	Undetermined	Yes
A-108	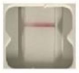	Undetermined	Undetermined	Yes

Sample ID	BinaxNow™	N1 Cт	N2 Cт	Consensus
A-109	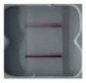	13.29	13.51	Yes
A-110	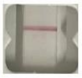	Undetermined	Undetermined	Yes
B-1	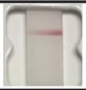	Undetermined	Undetermined	Yes
B-2	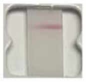	Undetermined	Undetermined	Yes
B-3	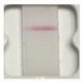	Undetermined	Undetermined	Yes
B-4	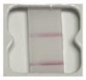	15.74	15.91	Yes
B-5	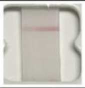	20.67	21.04	No
B-6	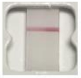	Undetermined	Undetermined	Yes
B-7	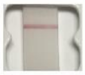	Undetermined	Undetermined	Yes
B-8	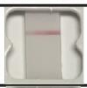	Undetermined	Undetermined	Yes
B-9	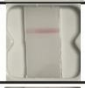	Undetermined	38.61	No
B-10	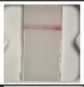	Undetermined	Undetermined	Yes
B-11	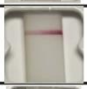	Undetermined	Undetermined	Yes
B-12	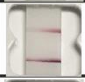	11.86	12.30	Yes
B-13	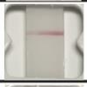	Undetermined	Undetermined	Yes
B-14	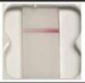	Undetermined	Undetermined	Yes
B-15	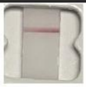	36.35	36.68	No
B-16	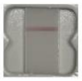	Undetermined	Undetermined	Yes

Sample ID	BinaxNow™	N1 Cт	N2 Cт	Consensus
B-17	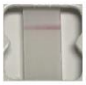	Undetermined	Undetermined	Yes
B-18	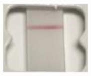	Undetermined	Undetermined	Yes
B-19	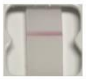	Undetermined	Undetermined	Yes
B-20	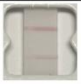	18.30	19.30	Yes
B-21	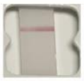	Undetermined	Undetermined	Yes
B-22	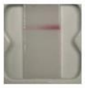	Undetermined	Undetermined	Yes
B-23	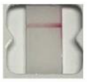	Undetermined	Undetermined	Yes
B-24	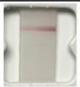	Undetermined	Undetermined	Yes
B-25	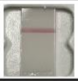	Undetermined	Undetermined	Yes
B-26	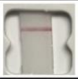	Undetermined	Undetermined	Yes
B-27	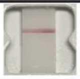	Undetermined	Undetermined	Yes
B-28	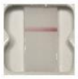	Undetermined	Undetermined	Yes
B-29	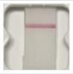	Undetermined	Undetermined	Yes
B-30	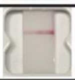	Undetermined	Undetermined	Yes
B-31	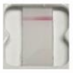	Undetermined	Undetermined	Yes
B-32	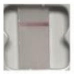	Undetermined	Undetermined	Yes
B-33	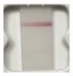	24.95	24.63	Yes
B-34	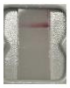	Undetermined	Undetermined	Yes

Sample ID	BinaxNow™	N1 Cт	N2 Cт	Consensus
B-35	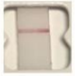	37.35	38.49	No
B-36	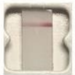	25.79	25.20	No
B-37	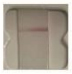	27.74	27.95	No
B-38	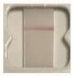	Undetermined	Undetermined	Yes
B-39	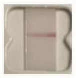	Undetermined	Undetermined	Yes
B-40	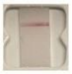	Undetermined	Undetermined	Yes
B-41	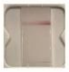	Undetermined	Undetermined	Yes
B-42	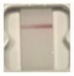	Undetermined	Undetermined	Yes
B-43	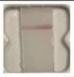	Undetermined	Undetermined	Yes
B-44	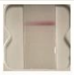	32.69	33.14	No
B-45	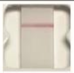	Undetermined	Undetermined	Yes
B-46	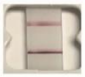	12.44	12.96	Yes
B-47	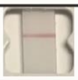	Undetermined	Undetermined	Yes
B-48	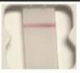	24.20	24.75	No
B-49	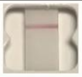	Undetermined	Undetermined	Yes
B-50	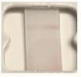	Undetermined	Undetermined	Yes
B-51	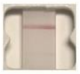	15.73	15.99	Yes
B-52	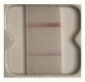	17.18	18.13	Yes
B-53	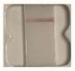	Undetermined	Undetermined	Yes
B-54	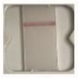	Undetermined	Undetermined	Yes
B-55	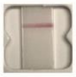	Undetermined	Undetermined	Yes
B-56	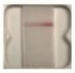	Undetermined	Undetermined	Yes
B-57	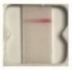	Undetermined	Undetermined	Yes
B-58	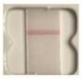	Undetermined	Undetermined	Yes
B-59	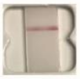	Undetermined	Undetermined	Yes
B-60	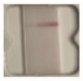	Undetermined	Undetermined	Yes
B-61	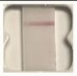	36.75	Undetermined	No
B-62	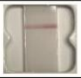	Undetermined	Undetermined	Yes
B-63	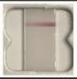	Undetermined	Undetermined	Yes
B-64	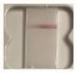	Undetermined	Undetermined	Yes
B-65	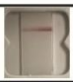	Undetermined	Undetermined	Yes
B-66	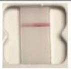	Undetermined	Undetermined	Yes
B-67	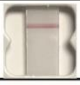	Undetermined	Undetermined	Yes
B-68	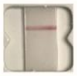	Undetermined	Undetermined	Yes
B-69	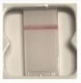	Undetermined	Undetermined	Yes
B-70	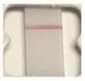	Undetermined	Undetermined	Yes

Sample ID	BinaxNow™	N1 CT	N2 CT	Consensus
B-71	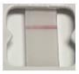	Undetermined	Undetermined	Yes
B-72	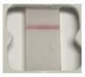	Undetermined	Undetermined	Yes
B-73	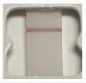	Undetermined	Undetermined	Yes
B-74	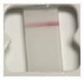	Undetermined	Undetermined	Yes
B-75	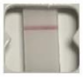	Undetermined	Undetermined	Yes
B-76	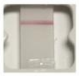	Undetermined	Undetermined	Yes
B-77	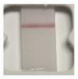	Undetermined	Undetermined	Yes
B-78	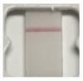	Undetermined	Undetermined	Yes
B-79	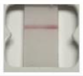	Undetermined	Undetermined	Yes
B-80	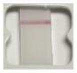	Undetermined	Undetermined	Yes
B-81	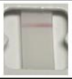	Undetermined	Undetermined	Yes
B-82	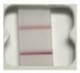	19.23	17.54	Yes
B-83	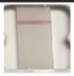	Undetermined	Undetermined	Yes
B-84	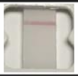	Undetermined	Undetermined	Yes
B-85	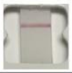	Undetermined	Undetermined	Yes
B-86	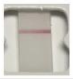	Undetermined	Undetermined	Yes
B-87	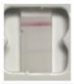	18.90	18.92	Yes
B-88	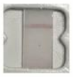	Undetermined	Undetermined	Yes
B-89	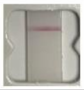	Undetermined	Undetermined	Yes
B-90	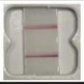	15.68	15.90	Yes
B-91	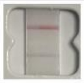	23.02	23.01	Yes
B-92	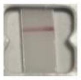	Undetermined	Undetermined	Yes
B-93	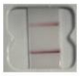	17.01	17.29	Yes
B-94	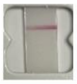	Undetermined	Undetermined	Yes
B-95	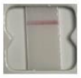	25.69	25.59	No
B-96	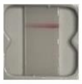	Undetermined	Undetermined	Yes
B-97	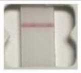	Undetermined	Undetermined	Yes
B-98	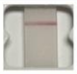	Undetermined	Undetermined	Yes
B-99	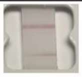	20.42	20.29	Yes
B-100	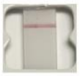	Undetermined	Undetermined	Yes
B-101	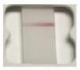	Undetermined	Undetermined	Yes
B-102	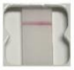	Undetermined	Undetermined	Yes
B-103	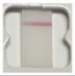	Undetermined	Undetermined	Yes
B-104	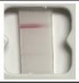	19.00	18.72	Yes
B-105	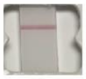	Undetermined	Undetermined	Yes
B-106	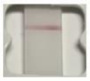	Undetermined	Undetermined	Yes

Sample ID	BinaxNow™	N1 C_T	N2 C_T	Consensus
B-107	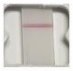	Undetermined	Undetermined	Yes
B-108	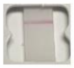	Undetermined	41.52	Yes
B-109	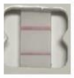	15.11	15.76	Yes
B-110	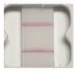	17.29	17.21	Yes
B-111	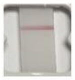	Undetermined	Undetermined	Yes
B-112	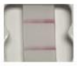	16.11	16.33	Yes
B-113	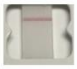	Undetermined	Undetermined	Yes
B-114	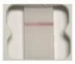	Undetermined	Undetermined	Yes
B-115	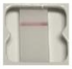	Undetermined	Undetermined	Yes
B-116	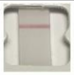	Undetermined	Undetermined	Yes
B-117	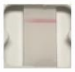	Undetermined	Undetermined	Yes
B-118	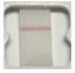	Undetermined	Undetermined	Yes
B-119	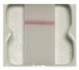	Undetermined	Undetermined	Yes
B-120	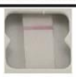	Undetermined	Undetermined	Yes
B-121	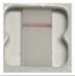	Undetermined	Undetermined	Yes
B-122	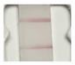	16.91	18.39	Yes
B-123	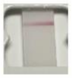	Undetermined	Undetermined	Yes
B-124	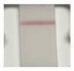	Undetermined	Undetermined	Yes

Sample ID	BinaxNow™	N1 C_T	N2 C_T	Consensus
B-125	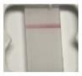	Undetermined	Undetermined	Yes
B-126	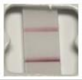	19.62	17.18	Yes
B-127	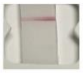	28.34	26.66	No
B-128	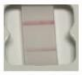	20.57	18.21	Yes
B-129	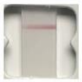	Undetermined	Undetermined	Yes
B-130	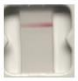	Undetermined	Undetermined	Yes
B-131	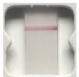	Undetermined	Undetermined	Yes
B-132	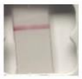	Undetermined	Undetermined	Yes
B-133	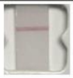	Undetermined	Undetermined	Yes
B-134	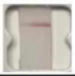	Undetermined	Undetermined	Yes
B-135	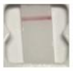	36.25	34.86	No
B-136	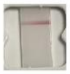	Undetermined	Undetermined	Yes
B-137	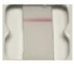	31.24	30.67	No
B-138	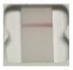	Undetermined	Undetermined	Yes
B-139	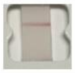	Undetermined	Undetermined	Yes
B-140	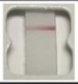	Undetermined	Undetermined	Yes
B-141	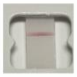	Undetermined	Undetermined	Yes
B-142	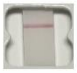	Undetermined	Undetermined	Yes

Sample ID	BinaxNow™	N1 C_T	N2 C_T	Consensus
B-143	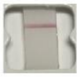	Undetermined	Undetermined	Yes
B-144	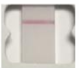	Undetermined	Undetermined	Yes
B-145	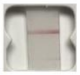	18.90	18.67	Yes
B-146	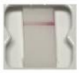	Undetermined	Undetermined	Yes
B-147	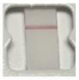	Undetermined	Undetermined	Yes
B-148	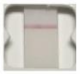	Undetermined	Undetermined	Yes
B-149	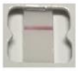	31.17	30.97	No
B-150	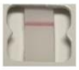	Undetermined	Undetermined	Yes
B-151	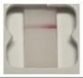	Undetermined	Undetermined	Yes
B-152	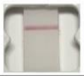	23.64	23.21	No
B-153	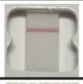	Undetermined	Undetermined	Yes
B-154	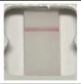	Undetermined	Undetermined	Yes
B-155	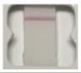	Undetermined	Undetermined	Yes
B-156	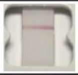	28.83	28.41	No
B-157	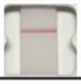	Undetermined	Undetermined	Yes
B-158	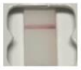	Undetermined	Undetermined	Yes
B-159	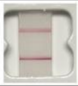	15.97	15.71	Yes
B-160	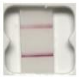	15.06	14.34	Yes

Sample ID	BinaxNow™	N1 C_T	N2 C_T	Consensus
B-161	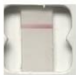	Undetermined	Undetermined	Yes
B-162	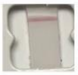	Undetermined	Undetermined	Yes
B-163	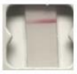	Undetermined	Undetermined	Yes
B-164	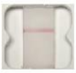	Undetermined	Undetermined	Yes
B-165	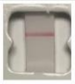	Undetermined	Undetermined	Yes
B-166	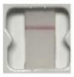	Undetermined	Undetermined	Yes
B-167	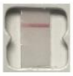	Undetermined	Undetermined	Yes
B-168	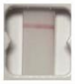	28.96	29.40	No
B-169	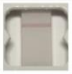	27.09	27.87	No
B-170	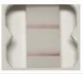	17.71	18.69	Yes
B-171	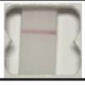	Undetermined	Undetermined	Yes
B-172	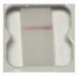	Undetermined	Undetermined	Yes
B-173	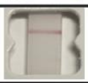	24.43	25.64	Yes
B-174	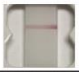	Undetermined	Undetermined	Yes
B-175	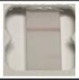	17.67	18.15	Yes
B-176	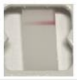	Undetermined	Undetermined	Yes
B-177	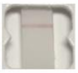	Undetermined	Undetermined	Yes
B-178	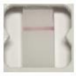	37.76	38.26	No

Sample ID	BinaxNow™	N1 Cт	N2 Cт	Consensus
B-179	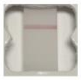	Undetermined	Undetermined	Yes
B-180	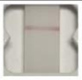	Undetermined	Undetermined	Yes
B-181	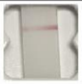	Undetermined	Undetermined	Yes
B-182	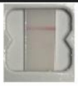	Undetermined	Undetermined	Yes
B-183	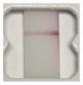	Undetermined	Undetermined	Yes
B-184	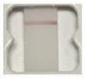	Undetermined	Undetermined	Yes
B-185	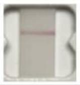	Undetermined	Undetermined	Yes
B-186	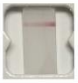	Undetermined	Undetermined	Yes
B-187	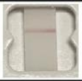	38.97	Undetermined	No
B-188	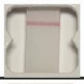	Undetermined	Undetermined	Yes
B-189	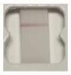	Undetermined	Undetermined	Yes
B-190	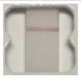	Undetermined	Undetermined	Yes
B-191	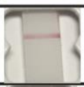	Undetermined	Undetermined	Yes
B-192	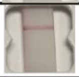	Undetermined	Undetermined	Yes
B-193	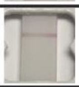	35.11	Undetermined	No
B-194	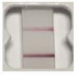	16.96	18.42	Yes
B-195	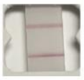	16.54	16.79	Yes
B-196	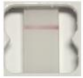	32.28	Undetermined	No

Sample ID	BinaxNow™	N1 Cт	N2 Cт	Consensus
B-197	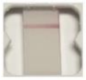	Undetermined	Undetermined	Yes
B-198	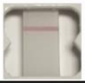	Undetermined	Undetermined	Yes
B-199	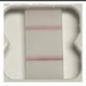	15.14	14.18	Yes
B-200	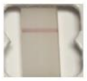	Undetermined	Undetermined	Yes
B-201	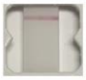	Undetermined	Undetermined	Yes
B-202	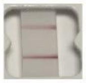	16.98	15.57	Yes
B-203	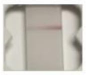	Undetermined	Undetermined	Yes
B-204	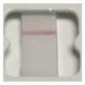	Undetermined	Undetermined	Yes
B-205	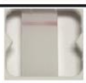	Undetermined	Undetermined	Yes
B-206	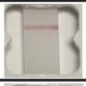	35.95	38.98	No
B-207	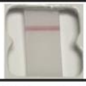	Undetermined	Undetermined	Yes
B-208	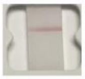	36.90	Undetermined	No
B-209	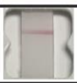	Undetermined	Undetermined	Yes

Supplemental Table 1. PANGO family assignments for positive samples. Successfully sequenced samples were assigned lineages with PANGO v4.0.6 and PANGO data v1.9. A summary of the main Omicron sublineages (B.1.1.529 and BA.X) observed are shown here.

PANGO family	Count (%)
B.1.1.529	7 (7.6%)
BA.1	70 (76.1%)
BA.2	15 (16.3%)

Supplemental Table 2. Nucleoprotein mutations in Omicron.

Nt change	AA change	Omicron sub-lineages	Instances	% of expected
28311 C>T	N P13L	B.1.1.529, BA.1, BA.2	92	100%
28361 GGAGAACGCA>G	N del31/33	B.1.1.529, BA.1, BA.2	92	100%
28881 G>A	N R203K	B.1.1.529, BA.1, BA.2	92	100%
28883 G>C	N G204K	B.1.1.529, BA.1, BA.2	91	98%
29510 A>C	N S413R	BA.2	22	147%

Supplemental Table 3. Mean and Standard Deviation of qRT-PCR positive by BinaxNOW™ result and targets

Test Period 1			
**Target**	**BinaxNOW™**	**Mean C_T**	**Standard Deviation**
N1	Positive	17.7	4.0
	Negative	31.5	4.4
N2	Positive	18.2	3.9
	Negative	31.5	4.0
**Test Period 2**			
**Target**	**BinaxNOW™**	**Mean C_T**	**Standard Deviation**
N1	Positive	17.7	2.9
	Negative	31.3	5.2
N2	Positive	17.7	2.9
	Negative	30.6	5.6
**Combined**			
**Target**	**BinaxNOW™**	**Mean C_T**	**Standard Deviation**
N1	Positive	17.7	3.4
	Negative	31.4	4.8
N2	Positive	17.9	3.4
	Negative	31.1	4.8

Supplemental Table 4. 2x2 Table for qRT-PCR and BinaxNOW™ matched samples using the Pilarowski, *et al.* classification (5).

**Pilarowski, *et al.* Classification**			
**Combined**			
	C_t ≤30	C_t >30	
	**RT-PCR +**	**RT-PCR -**	Totals
**Binax +**	55	0	55
**Binax -**	18	246	264
Totals	73	246	319

Supplemental Table 5. 2x2 Table for qRT-PCR and BinaxNOW™ combined matched samples with maximum Youden Index classification (25,26).

**ROC Curve Maximum Youden Index (0.98) Classification**			
**Combined**			
	C_t <24	C_t >24	
	**RT-PCR +**	**RT-PCR -**	Totals
**Binax +**	51	4	55
**Binax -**	3	261	24
Totals	54	265	319
